# Treatment of Intravenous Leiomyomatosis with Cardiac Extension following Incomplete Resection

**DOI:** 10.1155/2015/756141

**Published:** 2015-12-10

**Authors:** Mathew P. Doyle, Annette Li, Claudia I. Villanueva, Sheen C. S. Peeceeyen, Michael G. Cooper, Kevin C. Hanel, Gary G. Fermanis, Greg Robertson

**Affiliations:** ^1^Department of Cardiothoracic Surgery, St George Hospital, Sydney, NSW 2217, Australia; ^2^Graduate School of Medicine, University of Wollongong, Sydney, NSW 2252, Australia; ^3^University of New South Wales, Sydney, NSW 2000, Australia; ^4^Department of Anesthesia, St George Hospital, Sydney, NSW 2217, Australia; ^5^Department of Vascular Surgery, St George Hospital, Sydney, NSW 2217, Australia; ^6^Department of Obstetrics & Gynecology, St George Hospital, Sydney, NSW 2217, Australia

## Abstract

*Aim*. Intravenous leiomyomatosis (IVL) with cardiac extension (CE) is a rare variant of benign uterine leiomyoma. Incomplete resection has a recurrence rate of over 30%. Different hormonal treatments have been described following incomplete resection; however no standard therapy currently exists. We review the literature for medical treatments options following incomplete resection of IVL with CE.* Methods*. Electronic databases were searched for all studies reporting IVL with CE. These studies were then searched for reports of patients with inoperable or incomplete resection and any further medical treatments. Our database was searched for patients with medical therapy following incomplete resection of IVL with CE and their results were included.* Results*. All studies were either case reports or case series. Five literature reviews confirm that surgery is the only treatment to achieve cure. The uses of progesterone, estrogen modulation, gonadotropin-releasing hormone antagonism, and aromatase inhibition have been described following incomplete resection. Currently no studies have reviewed the outcomes of these treatments.* Conclusions*. Complete surgical resection is the only means of cure for IVL with CE, while multiple hormonal therapies have been used with varying results following incomplete resection. Aromatase inhibitors are the only reported treatment to prevent tumor progression or recurrence in patients with incompletely resected IVL with CE.

## 1. Introduction

Intravenous leiomyomatosis (IVL) with cardiac extension (CE) is a rare variant of otherwise usually benign uterine leiomyoma. Complete surgical excision is the only means of curative treatment. However this may not be technically feasible or individually desirable from the patient's perspective with combined thoracoabdominal operation usually required to achieve complete resection. Given the high recurrence rate following incomplete surgical resection, the use of adjuvant hormonal therapy has been investigated in an attempt to control or eradicate remnant disease. These agents have been used with varying success in patients with incompletely resected disease. This review discusses the use of hormonal treatment of IVL with CE, with two cases demonstrating the effective use of aromatase inhibitor therapy in both the short and long term when complete surgical resection is not achieved.

## 2. Methods

Electronic databases were searched for the terms “intravenous leiomyoma” OR “intravenous leiomyomatosis” OR “intravascular leiomyoma” OR “intravascular leiomyomatosis” AND “cardiac extension” OR “extension to the heart” OR “intracardiac” OR “intracardiac extension”. These results were then individually searched for cases of hormonal or medical treatment following incomplete resection. The term “benign metastasizing leiomyoma” (BML) was also included in our literature search as this pathology is similar to IVL and some authors describe treatments for both BML and IVL in their series. Each article was then individually reviewed to confirm cases or reports of cardiac extension of IVL. The references of the articles retrieved were also reviewed in order to identify any further relevant studies. All studies including case reports, case series, and original articles were included. Studies were limited to those published in the English language.

The cardiothoracic database at our institution was searched for any patients who had undergone sternotomy for resection of intravenous tumors with cardiac extension, from the inception of our unit database to the present. The cases identified had their pathology reports checked to identify the presence of IVL with CE. The medical records of these patients were accessed to identify the degree of surgical resection and any treatments given following incomplete resection. Outcomes were determined from specialist follow-up reports.

## 3. Results

All articles identified were case reports or case series. All available reports of hormonal therapy for IVL with CE are included in this review. Two articles reported on both BML and included cases of IVL in their reports. Five articles included literature reviews of IVL with CE. These studies discuss diagnosis, treatment, in particular surgical strategies, and outcomes. Hormonal therapies have included progesterone, estrogen modulators, hypothalamic-pituitary axis suppression of gonadotropin-releasing hormone, and estrogen antagonism. While the use of these hormonal agents as therapy following incomplete surgical resection is discussed in individual studies, there is currently no study investigating the outcomes for each individual hormonal agent.

Two cases were identified from our records of incomplete resection of IVL with CE. Both patients received hormonal therapy following surgical treatment. They are included in this review as their results contribute significantly to the available literature.

### 3.1. Case  1

**Figure 1:**
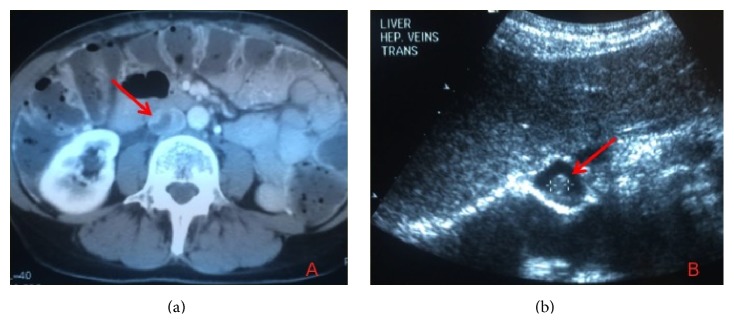
(a) Hypodense filling defect in the IVC on CT and (b) free-floating echodensity at the level of the hepatic veins, both consistent with intracaval thrombus.

A 53-year-old female was reported to have a free-floating thrombus in her inferior vena cava (IVC) identified on computed tomography (CT) of the abdomen, eight weeks following a right oophorectomy for an ovarian mass. The thrombus extended from the right external iliac vein to the level of the hepatic veins (Figure 1). She had undergone a hysterectomy and left oophorectomy eight years earlier for a uterine fibroid and left ovarian cyst. A diagnosis of IVL was established on her pathology specimen at that time; however the patient was lost to follow-up and no further action was taken. Following this new finding on CT, she underwent a combined sternolaparotomy with cardiopulmonary bypass (CPB) and ten-minute deep hypothermic circulatory arrest (DHCA). The IVC was opened and a smooth, firm white-colored tumor was resected with no need for further cardiotomy. She recovered uneventfully and was seen in outpatient follow-up 10 days later with no postoperative problems. Histopathology identified a 130 mm long rubbery cream tumor, with smooth muscle proliferation, variable cellularity, and organized thrombus, consistent with IVL. Specifically there were no features to warrant designation as borderline or malignant smooth muscle tumor and all features were typical of leiomyoma. During follow-up she developed bilateral pulmonary lesions that were not amenable to biopsy. She was treated with Tamoxifen 20 mg daily based on the strong estrogen and progesterone receptor positivity of her initial tumor. The lung lesions were stable following three months of therapy; however at 12 months her therapy was changed to Anastrozole as the nodules had progressed in size. 11 years following the commencement of Anastrozole the lung nodules remained stable and the patient remains asymptomatic.

### 3.2. Case  2

**Figure 2:**
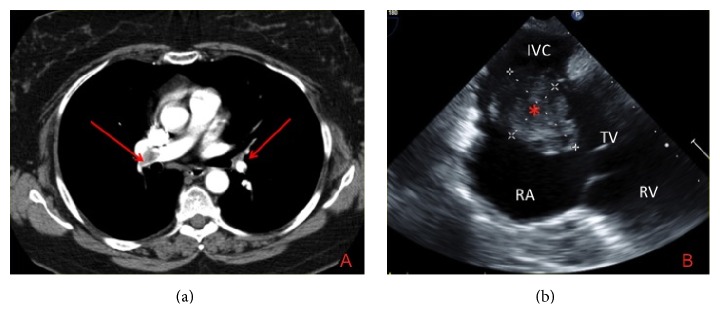
(a) Right and left pulmonary artery filling defects on CTPA and (b) TOE demonstrating the head of the mass protruding from the IVC into the RA. The TV was not damaged although the mass protruded through the TV into the RV during diastole. IVC: inferior vena cava; RA: right atrium; TV: tricuspid valve; RV: right ventricle.

**Figure 3:**
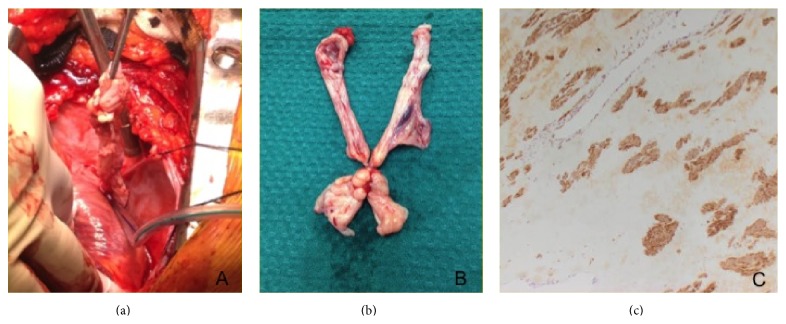
(a) A thin, white mass in the RA with its attachment extending down the IVC and (b) the gross specimen of the intracardiac tumor. (c) Smooth muscle actin antibody stain positive in the tumor specimen.

**Figure 4:**
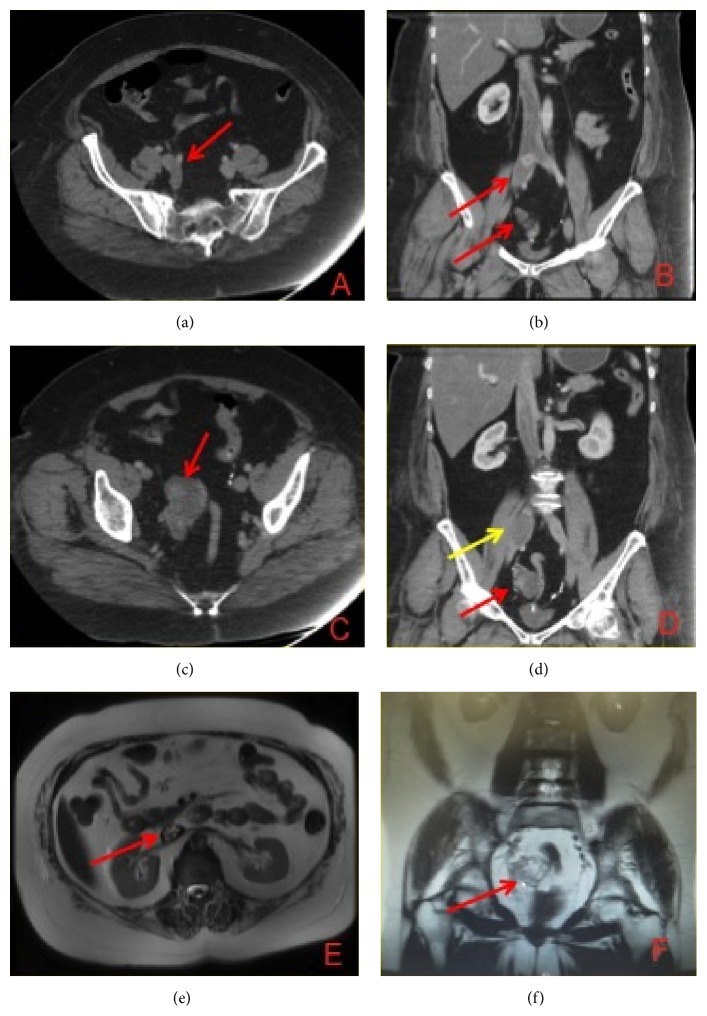
(a) CT axial and (b) coronal views of the IVL in the right common iliac vein (red arrows). (c) Axial and (d) coronal views of the pelvic component of the tumor (red arrows), abutting the dilated common iliac vein (yellow arrow). (e) Axial T2-weighted sequence on MRI identifying the intraluminal caval tumor with (f) associated pelvic mass on coronal image.

**Figure 5:**
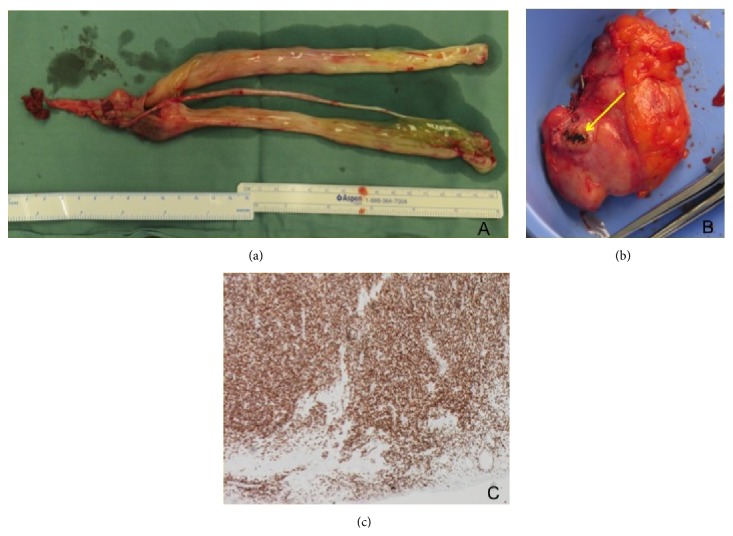
(a) Gross specimen of the tumor resected from the IVC venotomy during second-stage procedure and (b) pelvic mass with venous attachment site (yellow arrow) with (c) strongly positive staining of IVC tumor specimen for estrogen receptors.

A 62-year-old female was found to have bilateral pulmonary emboli on computed tomography pulmonary angiography (CTPA) following two episodes of syncope (Figure 2(a)). She underwent transthoracic echocardiography (TTE) and a large, mobile thrombus was reported in her right atrium (RA) and IVC (Figure 2(b)). She underwent emergency removal of the intracardiac mass and a large, white tumor mass protruded through the atriotomy when the right atrium was opened for venous cannulation for cardiopulmonary bypass (Figure 3(a)). The right atriotomy was extended and the mass was well visualized and divided at its apparent attachment to the IVC (Figure 3(b)). Histopathology identified a low-cellularity fibrous tumor with smooth muscle cells, with no areas of necrosis or atypia. Immunostaining was positive for smooth muscle actin and desmin, confirming the diagnosis of IVL (Figure 3(c)). Further abdominal imaging with CT identified a distended IVC, right common iliac vein, and right internal iliac vein, filled with low-density intraluminal material and a soft tissue density mass in the pelvis (Figure 4). She underwent a combined procedure with gynecologic oncology and vascular, general, and cardiothoracic surgery, all present. Redo-sternotomy and laparotomy were performed, the IVC was opened after gaining control of the proximal and distal vessels, and a white, firm, rubbery tumor nearly 30 cm long was excised with gentle traction (Figure 5(a)). The internal iliac vein had tumor remnant in it that was not resectable and the vein was ligated. The pelvic mass was identified abutting but not invading the sigmoid colon and was excised (Figure 5(b)). Histopathology identified identical material to the original specimen with strong estrogen and progesterone receptor positivity on immunostaining (Figure 5(c)). Her postoperative recovery was complicated by deep venous thrombosis of her right lower limb, treated with warfarin. Her recovery was otherwise unremarkable and she was commenced on Anastrozole 1 mg daily as per oncologic recommendation. She continues to remain well six months following her hospital discharge with ongoing oncology follow-up.

## 4. Discussion

Uterine leiomyoma is the most common benign pelvic tumor in women [1, 2]. Direct intravascular growth can occur in up to 30% of cases, with intravenous leiomyomatosis (IVL) defined as intravascular proliferation of benign-appearing smooth muscle tumor in the absence of, or beyond the confines of, a leiomyoma [3]. Almost all involved vessels are veins, or rarely lymphatics, and intra-arterial growth has not been described [3]. Any patient with intravenous invasion of leiomyoma identified either at initial surgical resection or on histological examination should be followed up with surveillance imaging, as these patients have the potential for propagation of the remnant tumor into the venous system. Cardiac extension (CE) is an uncommon sequelae of IVL, occurring in less than 10% of cases [4]. The smooth muscle cells proliferate and extend cranially up the inferior vena cava to the right heart, with potential to project into the right ventricle and pulmonary vasculature as a serpentine-like mass [5]. Intracaval growth is often asymptomatic until the right heart is reached [5–7]. Echocardiography is frequently first to identify the right-sided cardiac mass [5–7] and these lesions can be mistaken for primary cardiac tumors, most commonly myxoma [1–3, 5, 8]. Tumor embolism is very uncommon. This may be due to the dense fibromuscular and vascular composition of IVL which also lacks necrosis and hemorrhage [1, 2, 6]. Cardiac extension (CE) of IVL carries significant risk of morbidity and mortality, primarily from venous obstruction and cardiac insufficiency [4, 9–11].

Intravascular extension with cardiac involvement was first reported in 1907 [5, 12, 13]. Since that time, reports of IVL with CE have continued to increase in the literature [5–7, 14]. The majority of the current literature focuses on the presentation, risk factors, radiologic findings, surgical treatment, and outcomes [5–7, 15]. These reports clearly demonstrate that complete surgical resection should be the initial goal of therapy, as it offers almost certain cure [6, 7, 15]. However due to the indolent clinical course and often late presentation, as demonstrated by both our cases, many patients have extensive intravascular tumor that is not amenable to complete resection. The lung lesions that developed following resection of the intravascular tumor of our first case were diffuse and not accessible for resection or biopsy. Our second case had intravenous extension of IVL deep into the pelvis via the internal iliac vein, which was not amenable to safe resection, requiring ligation of the affected vein. Furthermore, some patients may choose not to undergo operative intervention. In light of these issues, the uses of medical treatments, which aim to reduce tumor volume or at least slow the progression of the tumor growth, have been trialed. The role of hormonal therapies for patients who cannot or are unwilling to have complete operative resection and the key principles for operative treatment of IVL with CE are discussed in this study.

### 4.1. Treatment: Surgery

The first successful operative resection of IVL with CE was reported in 1980 [16]. Since then multiple reports and reviews have discussed ideal operative technique for resection of IVL with CE [17–19]. Three conclusions can be drawn from the current literature in regard to surgical treatment. Firstly, complete surgical resection should be the goal of therapy as this offers almost certain cure and should occur promptly following discovery and diagnosis [6, 7]. Incomplete resection has a recurrence rate of up to 33% [6], with some authors reporting rapid regrowth following resection of the cardiac portion [20, 21]. The tumor can also recur following subtotal resection despite peripheral estrogen deprivation via medical therapy or total hysterectomy and bilateral salpingo-oophorectomy, further emphasizing the importance of complete excision where possible [15].

Secondly, the intracardiac portion of the tumor should be urgently resected if the patient presents with any signs of cardiac obstruction or failure [6, 7]. However, complete tumor excision from the chest alone should not be attempted, as reports of this approach have resulted in death from tearing the IVC and retroperitoneal hemorrhage [13, 22].

Finally, either a single- or multistage operative approach can be used with similar low intra- and postoperative complications. The urgency, surgical approach, and combination or separation of abdominal and thoracic procedures depend on the presentation, extent of the tumor, patient factors, and surgical services available [7, 23]. A staged procedure involves resecting the cardiac component of the tumor first, followed by an abdominal approach for the remaining IVC and pelvic tumor components [17, 24]. The second approach utilizes a single-stage abdominothoracic operation with a combined sternolaparotomy and cardiopulmonary bypass with or without deep hypothermic circulatory arrest [23]. Both have been performed with minimal operative morbidity and mortality, and either approach can be used depending on the extent of the pelvic and intravascular extent of the tumor [7]. More recently, a third approach has been described using a single-staged procedure utilizing an abdominal-only incision. These authors reported complete resection of the entire tumor via an abdominal IVC venotomy, without the use of cardiopulmonary bypass, or circulatory arrest [17]. While there are concerns about tumor embolization, the authors state that this risk is extremely low. Confident diagnosis preoperatively is required as other caval-infiltrating tumors such as renal-cell carcinoma have a much higher propensity for tumor fracture or embolization [17]. Regardless of approach, intraoperative transesophageal echocardiography should be performed to visualize the proximal end of the tumor and help guide complete resection [6, 7, 17].

### 4.2. Treatment: Medical Therapy

The use of hormonal therapy in IVL with CE was initially trialed on the theoretical basis that the tumor has similar hormonal characteristics to myometrial tissue and uterine leiomyoma [7]. If effective, hormonal treatment could slow disease progression and allow patients to continue with minimal symptoms following incomplete resection. It may also provide a treatment option for patients who refuse, or are unfit for, surgery. The other favorable characteristic for hormonal therapy in IVL with CE is that patients have almost uniformly undergone previous hysterectomy, so the consideration of fertility preservation in association with hormonal management is usually not required.

Hormonal therapies used in IVL with CE aim to induce a state of systemic hypoestrogenism, thereby starving tumor cells of their hormonal growth stimulus. Early reports of postoperative hormone therapy after complete surgical resection recommended against their use as side effects are common due to the systemic nature of hormonal treatment as well as the realization that complete surgical excision offered curative treatment. This meant that these treatments were no longer needed following surgery in most reported cases. Recent reviews of IVL with CE treatment state that the use of hormone treatments such as Tamoxifen, Letrozole, or Methoxyprogesterone does not offer survival benefits or prevent tumor recurrence following incomplete excision [6, 7]. Not all classes of available hormonal agents were scrutinized in this review though, and it is unclear about the follow-up of treated patients and whether they were adherent to treatment regimes. Our two cases demonstrate the potential for IVL to recur at the operative site as well as presenting as new disease in a remote site, most commonly the lungs. Both cases highlight the need for further treatment if the tumor cannot be confidently excised in entirety, regardless of the location of the residual tumor. It is clear from our first case that hormonal treatment can have varying effects on IVL with CE, depending on the class of agent used. Using aromatase inhibition provided our patient with disease-free progression and symptom-free daily life for over ten years, including having no reportable side effects of AI use at last known follow-up. Our second case highlights the benefits of single agent therapy with a relatively favorable side effect profile that can be safely used in patients following incomplete resection. This review discusses the use of all hormonal treatment described in the literature and their results in the treatment of IVL with CE.

#### 4.2.1. Progesterone

The first hormonal therapies for uterine leiomyoma were reported in the early 1980s. Leiomyoma expresses both estrogen and progesterone receptors, with estradiol able to induce progesterone receptor expression and support progesterone action on leiomyoma tissue [25]. In uterine leiomyoma, two studies using depot-medroxyprogesterone acetate (DMPA) demonstrated disease regression and improvement of symptoms [11, 26]. Evans and Cramer though reported increased lesion size and worsening of symptoms [9, 10]. Despite IVL displaying similar progesterone receptor characteristics to uterine lesions, there is little evidence surrounding the use of progesterone deprivation for the treatment of IVL. The development of targeted estrogen therapy has superseded the use of progesterone therapy, and there are no recent reports of progesterone use in treatment of IVL with CE.

#### 4.2.2. Selective Estrogen Receptor Modulators (SERMs)

SERMs are nonsteroidal estrogen receptor ligands that display tissue specific agonist-antagonist estrogenic action [25]. Leiomyomas express estrogen receptors and demonstrate increased growth during hyperestrogenic states [7]. Although initial results were favorable, the use of SERMs for treatment of uterine leiomyomas has not shown consistent evidence that this treatment reduces tumor size or improves clinical outcomes [27, 28]. However SERMs such as Tamoxifen and Raloxifene have been used as both monotherapy and adjuvant treatment for IVL with CE.

An analysis of 194 cases of IVL with CE concluded that postoperative antiestrogen therapy using Tamoxifen did not prevent tumor recurrence [6]. Studies were limited to case reports and series only. This recommendation is in keeping with the outcome of our first case, which developed pulmonary lesions following resection of the caval portion of her IVL with CE. Similarly, Lewis and colleagues reviewed the use of Tamoxifen in BML, which is histologically similar to IVL, with benign leiomyoma cells depositing in the lungs. This group found that Tamoxifen prevented disease progression in only 2 of 10 cases; the remaining 8 cases had disease progression [29].

In addition to these treatment results, SERMs such as Tamoxifen are also not recommended for use in women with a previous history of thrombotic events such as deep venous thrombosis (DVT), stroke, and pulmonary embolus (PE). Considering that many patients with IVL with CE present with symptoms suggesting PE or DVT, this treatment is not appropriate for these patients. The use of Tamoxifen in our first case essentially demonstrates a similar response to many other documented cases of its use in IVL with CE. It may have restricted the progression of the tumor initially; however this may also simply be attributable to the slow progressive nature of the tumor. After one year of treatment with Tamoxifen, new pulmonary lesions had developed and treatment was switched to an aromatase inhibitor. Tamoxifen also has a range of adverse effects, which contribute to the unsuitability of long-term antiestrogen therapy and were experienced by our first patient. Primarily, agonistic effects on the estrogen receptors of the endometrium increase the risk of endometrial carcinoma [25], as well as menopause induction, osteoporosis, and cardiovascular disease [28].

#### 4.2.3. Gonadotropin-Releasing Hormone (GnRH) Agonists

The use of GnRH agonists has been increasingly investigated for the treatment of uterine leiomyoma, as well as breast and prostate cancer. GnRH agonists are a synthetic peptide modeled after the hypothalamic neurohormone GnRH which interacts with the receptor to release pituitary hormones FSH and LH, causing systemic estrogen and progesterone suppression [30]. Through the direct, competitive inhibition of the pituitary GnRH receptor, GnRH agonists have been used to prevent recurrence of IVL with CE by inducing a state of hypoestrogenism [31, 32]. Hameleers and colleagues described treating a patient for a period of three years with a GnRH analogue following incomplete resection of IVL with CE. She had a small growth in her residual tumor but remained clinically asymptomatic [33]. Mitsuhashi and colleagues reported the use of the GnRH agonist leuprorelin acetate for six months following the incomplete resection of the pelvic component of IVL with CE, which was confirmed to be estrogen receptor positive. The tumor began to grow following the cessation of GnRH agonist therapy, and growth was inhibited once the therapy was recommenced [31]. Both Jung Choi and Morice describe the use of GnRH agonist following a single-stage resection of IVL with CE, both with a pelvic mass also requiring resection. No progression of disease was seen following six and ten months of treatment, respectively [4, 34].

The main concerning features of long-term therapy with GnRH agonists are a reduction in bone mineral density caused by low circulating estrogen levels [35, 36]. Other side effects associated with these hormonal changes include vasomotor symptoms such as hot flushes and headaches and an altered lipid profile predisposing to cardiovascular disease [36]. Long-term use has resulted in attempts to offset these effects through a technique of “adding back” steroid support through the use of other agents, including SERMs such as Raloxifene [36]. There are no reports of this application of therapy to IVL with CE.

#### 4.2.4. Aromatase Inhibitors (AIs)

Aromatase catalyzes the final step in estrogen synthesis, converting the androgens androstenedione and testosterone to the estrogens estrone and estradiol [37]. AIs are a class of antiestrogens that potently inhibit the aromatase enzyme in all tissues. AIs reversibly bind to the aromatase enzyme complex and suppress over 95% of tissue aromatase activity. This results in significant reductions in systemic circulating estrogen and has no effect on the formation of adrenal corticosteroids or aldosterone [37, 38]. First-generation AIs were used in estrogen-receptor positive postmenopausal breast cancer. Now, third-generation nonsteroidal inhibitors such as Anastrozole and Letrozole are most commonly used in clinical practice, remaining primarily in estrogen and progesterone-receptor positive breast cancer [39]. Leiomyoma also expresses strong ER positivity and AIs have been the focus of several studies looking at their effects on size, symptom improvement, and other outcomes such as arterial blood supply in uterine leiomyoma [40–45]. While the current levels of evidence are insufficient to provide strong clinical recommendations for the use of AIs in uterine fibroids [46], several articles have reported the potential advantages over other hormonal treatments [40–43]. Parsanezhad et al. directly compared the 3rd-generation AI Letrozole to the GnRH agonist Triptorelin in a randomized controlled trial investigating their effects on leiomyoma volume and hormonal status [42]. They demonstrated a trend towards greater uterine leiomyoma volume reduction with AI treatment compared to a GnRH agonist, with a much more favorable side effect profile. They also commented that AI use did not alter systemic hormonal profile. Gurates and Hilário both demonstrated that AIs had a minimal side effect profile and reduced the size and symptoms of uterine leiomyoma [40, 41]. Brito et al. demonstrated in a small study that there was no change in uterine blood flow with the use of AIs in uterine leiomyoma, suggesting that the effects of the therapy are strictly based on the hormonal blockade and not vascular in nature [43]. AIs are particularly useful in leiomyoma treatment based on this finding, as leiomyoma cells produce their own estrogen, with AIs inhibiting the estrogen production from these cells as well as normal ovarian and adipose cells [47–49]. Lewis et al. discuss a case of IVL with CE and pulmonary lesions who underwent resection of the cardiac portion of her tumor only, followed by treatment with an AI (Anastrozole) with initial symptomatic improvement. The patient ceased her AI treatment and represented with symptom recurrence from her IVC mass. While the pathology is similar but not identical, BML has 5 case reports in the literature of AI use. All patients had stable or improved disease on follow-up [29, 50].

Only a single case report for AI use in IVL with CE is available in the current literature. This report by Biri and colleagues describe the use of Letrozole in a 39-year-old woman who had pelvic recurrence of incompletely resected IVL [51]. After 6 months of treatment, repeat imaging showed no progression of her pelvic disease.

Our two cases represent the only other reports of AI use in IVL with CE and the first reports of the use of Anastrozole specifically. In our two cases, the tumors of both patients had strong estrogen receptor positivity confirmed on histological staining. While long term follow-up is not yet available for our second case, Anastrozole use has proven to be an effective therapy in our first case, with no tumor growth of either lung nodules or intravenous tumor recurrence over a ten-year period.

Bone mineral density loss and vasomotor symptoms such as flushes are the most commonly reported side effects of AIs. However, these are reported less frequently than with GnRH use [42]. The mechanism for this is not completely understood; however it is most likely due to the maintenance of near-physiologic systemic hormone profile. Our second case underwent bone mineral densitometry prior to commencing her anastrozole therapy and prophylactically commenced on oral vitamin-D supplementation.

## 5. Conclusion

Surgical excision of IVL with CE offers curative treatment and can be performed using either a single- or multistaged operative approach. If complete resection is not achieved ongoing treatment with hormonal agents should be considered as the recurrence rate can be over 30%. The use of hormonal therapies traditionally used in uterine leiomyoma for suppression of the estrogen-sensitive growth of IVL with CE has yielded mixed results. Aromatase inhibitors are a relatively recent treatment addition to the hormonal blockade of estrogen-dependent tumors. These agents can potentially provide patients with incompletely resected IVL with CE, with progression-free survival in the short to medium term.
